# Unstimulated salivary flow, pH, proteins and oral health in patients with Juvenile Idiopathic Arthritis

**DOI:** 10.1186/s12903-017-0386-1

**Published:** 2017-06-02

**Authors:** Agnieszka Kobus, Anna Kierklo, Anna Zalewska, Anna Kuźmiuk, Sławomir Dariusz Szajda, Sławomir Ławicki, Joanna Bagińska

**Affiliations:** 1 Department of Dentistry Propaedeutics, Medical University of Bialystok, ul. Waszyngtona 15A, 15-274 Bialystok, Poland; 2 Department of Conservative Dentistry, Medical University of Bialystok, ul. M. Sklodowskiej-Curie 24A, 15-274 Bialystok, Poland; 3 Department of Pediatric Dentistry, Medical University of Bialystok, ul. Waszyngtona 15A, 15-274 Bialystok, Poland; 4 Department of Psychiatry, Medical University of Bialystok, Plac Brodowicza 1, 16-070 Choroszcz, Poland; 5 Department of Biochemical Diagnostics, Medical University of Bialystok, ul. M. Sklodowskiej-Curie 24A, 15-274 Bialystok, Poland

**Keywords:** Juvenile Idiopathic Arthritis, Saliva proteins, Salivary flow rate, DMFT, Oral health, Oral hygiene

## Abstract

**Background:**

There have been inconsistent conclusions regarding salivary abnormalities and their effect on oral health of Juvenile Idiopathic Arthritis (JIA) patients. The purpose of the study was to evaluate the flow rate and selected biochemical parameters of unstimulated whole saliva in correlation to oral health in JIA children.

**Methods:**

Thirty-four JIA patients and 34 age- and sex-matched controls not affected by JIA (C) were divided into two groups: with mixed and permanent dentition. DMFT/dmft, gingival and simplified oral hygiene indices were evaluated. Salivary flow rate, pH, lysozyme, lactoferrin, salivary protein concentrations and peroxidase activity were assessed.

**Results:**

The salivary flow rate was significantly lower in the total JIA group (0.41 ml/min) as compared with the C (0.51 ml/min) and in the permanent dentition of JIA children (0.43 ml/min) as compared with the C (0.61 ml/min). A significantly lower pH was observed in total (6.74), mixed (6.7) and permanent (6.76) dentition of JIA groups in comparison to the C (7.25, 7.21, 7.28 respectively). The specific activity of peroxidase was significantly higher in JIA patients (total 112.72 IU/l, mixed dentition 112.98 IU/l, permanent dentition 112.5 IU/l) than in the C group (total 70.03 IU/l, mixed dentition 71.83 IU/l, permanent dentition 68.61 IU/l). The lysozyme concentration in JIA patients (total and permanent dentition groups) was significantly higher than in the C group. There were no significant differences in lactoferrin and salivary protein concentrations. There were no statistically significant differences in oral status between JIA patients and C, respectively: DMFT = 5.71, dmft = 3.73, OHI-S = 0.95, GI = 0.25 and DMFT 5.71, dmft = 3.73, OHI-S = 0.85, GI = 0.24. The specific activity of peroxidase in the unstimulated whole saliva was inversely correlated with the GI index, whereas the salivary lysozyme concentration was inversely correlated with the dmft index in JIA patients.

**Conclusion:**

In the course of JIA occur a reduction of the resting salivary flow rate and a decrease of saliva pH. In spite of this, no differences in the clinical oral status between the JIA children population and the control group were found. The mobilisation of salivary peroxidase and lysozyme contributes to the maintenance of healthy oral tissues.

## Background

Juvenile Idiopathic Arthritis (JIA) is an autoimmune heterogeneous disease with a spectrum of joint involvement and associated systemic involvement. This disease is characterized by joint inflammation with symptoms persisting for more than six weeks and onset before 16 years of age [1]. The worldwide prevalence of JIA ranges from 16 to 150 per 100 000 [2], with an annual incidence from 3.2 to 24 per 100 000 [3–8]. In Poland, the annual incidence of JIA is estimated to be 6.4 per 100 000 [9]. Currently, JIA is classified according to the International League of Associations for Rheumatology (ILAR) as: systemic arthritis, rheumatoid factor (RF) negative polyarthritis, RF-positive polyarthritis, oligoarthritis, enthesitis-related arthritis, psoriatic arthritis and undifferentiated arthritis [10]. Females are much more frequently affected with almost all types of JIA [11]. The aetiopathogenesis of JIA is not entirely understood, but abnormal immunoregulation, cytokine production, immunogenetic predisposition and latent viral infection may be involved [12].

Theoretically, there is a significant risk to oral health in JIA patients for several reasons. Synovial inflammation in JIA is usually accompanied by systemic signs of inflammation such as increased levels of acute-phase proteins in serum leading to joint cartilage and bone destruction [13, 14]. These alterations in bone growth may lead to impaired overall growth of the patients, and also to impaired mandibular growth, causing severe retrognathia, open bite (in which the anterior teeth have no contact during occlusion), microgenia and “bird like faces” [2]. The disease is therefore an important cause of short- and long-term functional disability of children [11]. The prevalence of temporomandibular joint (TMJ) dysfunction in JIA patients varies between 25% and 75% [15]. The involvement of the TMJ may result in restricted mouth opening, alterations in masticatory function and difficulties in maintenance of patients’ oral hygiene and dental treatment [16].

A second factor affecting oral health, narrowly increased incidence of dental caries and gingivitis, is the involvement of the upper limb (especially the hands) in JIA, causing a functional disability and difficulties with fine motor movements required for efficient tooth brushing and flossing, hence plaque removal [2, 17, 18].

Medications used to control the inflammation in JIA patients may also influence oral health. Sugar-based elixir forms of oral medications, mainly nonsteriodal anti-inflammatory drugs, often applied to young children, increase the risk of dental caries. Pill or tablet formulations are frequently unswallowable, therefore young patients may suck or chew them, which causes soft tissue ulceration and dental erosion [19]. The possible side effects of taking corticosteroids affecting the mouth include delayed wound healing and increased risk of infection [2, 19].

Saliva plays a crucial role in maintaining oral health. This heterogeneous fluid is composed of proteins, glycoproteins, electrolytes and small organic molecules transported from blood. The functions of saliva include buffering capacity, dental remineralisation, antimicrobial activity, lubrication, mucosa regeneration, articulation and food digestion [20]. The results of evaluation of salivary parameters in JIA patients are inconclusive, some studies showed a reduced resting salivary flow (SF) and a reduced response to stimulation [21–23], while other studies showed no differences in stimulated and unstimulated SF between subjects and healthy controls [24–26]. It was proved that JIA patients were characterized by altered saliva biochemistry, namely reduced antimicrobial proteins such as immunoglobulin A, lysozyme (Lz), reduced pH and reduced inorganic ions, particularly potassium, calcium and phosphorus [23, 24, 26]. All above mentioned salivary biochemical abnormalities may contribute to the increased risk of caries and gingivitis in individuals suffering from JIA. However, some studies on JIA children have shown a significantly higher salivary antioxidant activity, namely peroxidase (OPO) activity and significantly lower metalloproteinase levels [27, 28].

We hypothesized that salivary abnormalities in JIA patients influenced intraoral health. The aim of the present study was to evaluate the flow rate and selected biochemical parameters of unstimulated whole saliva (UWS) in correlation to oral health in JIA children and controls not affected by JIA including mixed and permanent dentition.

## Methods

The study was approved by the Bioethical Committee of the Medical University of Bialystok, Poland (No. R-I-002/53/2008 and No. R-I-002/494/2015). An informed written consent was obtained from the parent(s) or guardian(s) and a verbal consent was given by the patients after explanation of the nature, purpose and potential risks of the study.

### Subjects

Patients treated at the Outpatient Clinic of the Department of Paediatrics and Developmental Disorders, Medical University of Bialystok, Poland, in the years 2009–2010 were included in the study. This Outpatient Clinic is the only one pediatric rheumatology clinic in North-Eastern Poland. The inclusion criterion was diagnosed JIA. The exclusion criteria for patients were: the presence of another chronic disease, the treatment with a drug that interferes with salivary secretion in the last year and for girls the onset of menstruation. The patients were examined by the same physician according to the ILAR classification [29]. Patients were recruited consecutively during the routine follow-up. The total number of patients invited to the study was 61, whereas 7 parents refused to participate and 20 patients were excluded. The patients were divided into two subgroups: with mixed dentition (MD) and with permanent dentition (PD).

### Controls

Children not affected by JIA (C), matched for year of birth, gender and ethnicity with the subjects, were recruited from a local dental office. At least two potential controls were identified for each subject and one of them was randomly chosen. In case of refusal or failure to meet the inclusion criteria the next child from the list was invited. Individuals participating in the study met each of the following eligibility criteria: good health and no systemic illness or hospitalization during last two years, no known history of chronic disease and no medications or hormones that interfere with salivary secretion in the last year and for girls the onset of menstruation. Finally, one control for each case was found.

### Saliva collection

The subjects were instructed to refrain from the consumption of food and beverages, except water, for two hours before saliva collection. For saliva collection, each participant was seated in a chair in a well-ventilated room and protected from gustatory and other stimulation. Resting whole saliva samples were collected to plastic tubes on ice for 15 min, under control of one dentist (AK), by passive spitting method, between 8:00 and 10:00 _AM_ to minimize the circadian rhythm effects [30, 31]. The volume of each sample was measured with a pipette calibrated in 0.1 ml units. SF was determined from the obtained volume divided by the time needed for sample collection. Immediately after saliva collection, pH was determined. After measurement of the volumes, salivary samples were centrifuged at 3000 × g for 20 min at 4 °C to remove cells and debris. The resulting supernatants were divided into 200 μl portions, frozen and kept at −80 °C until analyzed, with the exception of portions for OPO activity, which was determined immediately after centrifugation.

### Assays

#### pH

pH was measured using a pH meter (pH Jonometer CPI 501, Elmetron).

### Lactoferrin

An enzyme-linked immunosorbent assay (ELISA) method was used to determine the salivary lactoferrin (Lf) concentration using MaxiSorp^TM^ microplates (Nunc Brand cat. no 449824, Roskilde, Denmark) coated with rabbit anti human lactoferrin (primary) antibody 1:500 (Sigma, L3262, St Louis, MO) and blocked with 2% bovine serum albumin (BSA; Sigma, A7030) according to Waszkiewicz et al. [32]. The results were analyzed by the KC Junior programme (Bio-Tek software applications for MS-DOS), all manual and automated readers.

### Specific activity of peroxidase

Specific activity of OPO in unstimulated saliva was determined colometrically according to Mansson-Rahemtulla et al. [33].

### Lysozyme

The salivary Lz concentration in unstimulated saliva was determined by radial immunodiffusion (Human ‘NL’ Nanorid plate, The Binding Site, Birmingham, UK) as described by Mancini et al. [34]. 10 μl of appropriate diluted saliva, undiluted control serum and calibrators were placed into agarose gel immunodiffusion plates containing the monospecific antibody. The plates were then tightly closed, re-sealed in their original pouches and placed into the moist chamber. After 75 h of incubation at room temperature, the precipitation ring diameters were measured using the Digital RID Plate Reader and analysed using the RIDRead software. The Lz concentration was expressed in mg/l.

### Proteins

The salivary protein content (SP) was measured by bicinchoninic acid BCA method with bovine serum albumin as a standard (PIERCE BCA Protein Assay Kit, No 23225, Rockford, IL, USA) [35].

All analyses were performed in duplicate.

### Clinical assessment

All patients were asked about subjective oral dryness and angulitis, frequency of sweets consumption and the occurrence of occlusal abnormalities in the family. Clinical examinations were performed by one qualified dental surgeon (AK) under standardized conditions, in a dental chair using portable equipment with artificial light, suction device and compressed air. All examinations were carried out using diagnostic dental tools (plane mirror, clinical probe and periodontal probe). Following the World Health Organization criteria, the level of dental caries in subjects was determined using the DMFT index (Decayed, Missing or Filled teeth of the permanent dentition) in children with permanent and mixed teeth and using the dmft index (decayed, missing or filled teeth of the primary dentition) in children with mixed dentition [36]. The white spot lesions were excluded. The gingival status was determined using the gingival index (GI) [37]. The gingival status was coded as follows: 0- no gingivitis, above 0 to 1- mild gingivitis, above 1 to 2- moderate gingivitis, above 2 up to 3- severe gingivitis. The oral hygiene was determined with the use of the oral hygiene index – simplified (OHI-S) [38]. It was assumed that the value of the OHI-S index fluctuated between 0 and 6 where 0–2 means good oral hygiene, 2–4 satisfactory oral hygiene, and 4–6 bad oral hygiene.

Dental treatment was offered to all the examined patients, but only a small group of them accepted the proposal. A routine hygienic procedure, caries and preventive treatment were performed. In the group of patients with a high risk of bacteraemia the antibiotic protection was applied.

Prior to the study, a calibration for caries by double examination of 10 children aged between 6 and 15 was performed in the interval of one week. The intra-examiner agreement (unweighted Cohen’s kappa coefficient) was 0.89 for primary dentition and 0.92 for permanent dentition.

### Statistical analysis

The statistical analysis was performed using the Statistica version 10.0 (Statsoft, Cracow, Poland). The Mann–Whitney test was used to compare the difference in prevalence for quantitative values. The results were presented in a descriptive manner: mean ± SD, minimum and maximum values for continuous variables. The differences of children’s subjective complaints were assessed by the Fisher’s exact test. The Fisher-Freeman-Halton test was used to assess the differences of children’s subjective complaints in the case of the number of quality variable variants greater than 2 (consumption of sweets). The Spearman’s rank correlation coefficient was used to study the associations between salivary parameters (SF, pH, OPO, Lf, Lz, SP) and oral health condition (DMFT, dmft, GI, PBI). The statistical significance was defined as *p* ≤ 0.05.

## Results

The final study population consisted of thirty-four subjects with a medical diagnosis of JIA (aged 6 to 18 years, 64.7% of females) and a similar number of controls. The subgroup with mixed dentition (MD) consisted of 15 patients (aged 6 to 10 years) and the subgroup with permanent dentition (PD) of 19 patients (aged 11 to 18 years). Table 1 shows in detail the age and sex of the participants and the disease duration, including the type of dentition. The mean duration of the disease was 4.62 years. The dental and periodontal status and oral hygiene of the JIA patients and controls, including mixed and permanent dentition, are presented in Table 2. We found no differences in dental hygiene, dental and periodontal status between JIA children and control group.

**Table 1:** Demographic characteristics and disease duration in JIA children and controls (C) including mixed and permanent dentition

Demographic characteristic			Total		Mixed dentition		Permanent dentition	
			JIA *N =* 34	C *N =* 34	JIA *N =* 15	C *N =* 15	JIA *N =* 19	C *N =* 19
Age (Years)		Mean(SD)	12.29 (4.57)	12.64 (4.35)	7.47 (1.46)	8.53 (2.35)	15.95 (2.17)	15.89 (2.28)
		Min-Max	6–18	6–18	6–10	6–13	11–18	11–18
		*p-*value	0.74		0.384		1.0	
Sex	Female	Mean (%)	21 (61.76)	21 (61.76)	10 (66.67%)	10 (66.67%)	11 (57.90%)	11 (57.90%)
	Male	Mean (%)	13 (38.24)	13 (38.24)	5 (33.33)	5 (33.33)	8 (42.10)	8 (42.10)
Disease duration (Years)		Mean (SD)	4.62 (3.53)		3.15 (2.61)		5.71 (3.79)	
		Min-Max	0.17–13		0.25–8		0.17–13	

Using Mann–Whitney test

**Table 2:** Oral parameters in JIA children and controls (C) including mixed and permanent dentition

Variables		Total			Mixed dentition			Permanent dentition		
		JIA *N =* 34	C *N =* 34	*p-*value	JIA *N =* 15	C *N =* 15	*p-*value	JIA *N =* 19	C *N =* 19	*p-*value
DMFT	Number of caries free individuals	6	9		5	8		1	1	
	Mean (SD)	6.21 (5.49)	5.71 (5.33)	0.66	2 (2.36)	1.27 (1.67)	0.66	9.53 (4.96)	9.21 (4.54)	0.66
	Min-Max	0–19	0–17		0–7	0–5		0–19	0–17	
Decayed	Mean (SD)	1.94 (2.37)	2.47 (3.59)	0.69	0.53 (1.06)	0.2 (0.56)	0.23	3.05 (2.55)	4.26 (3.96)	0.55
	Min-Max	0–10	0–11		0–4	0–2		0–10	0–11	
Missing	Mean (SD)	0.23 (0.92)	0.06 (0.24)	0.61	0	0	1.0	0.42 (1.22)	0.1 (0.31)	0.57
	Min-Max	0–5	0–1		0	0		0–5	0–1	
Filled	Mean (SD)	4.03 (4.63)	3.18 (3.86)	0.62	1.47 (2.23)	1.07 (1.71)	0.66	6.04 (5.06)	4.84 (4.13)	0.62
	Min-Max	0–17	0–14		0–7	0–5		0–17	0–14	
dmft	Number of caries free individuals	1	0		1	0		-	-	
	Mean (SD)	5.47 (3.74)	3.73 (2.58)	0.21	5.47 (3.74)	3.73 (2.58)	0.21	-	-	
	Min-Max	0–12	0–8		0–12	0–8		-	-	
decayed	Mean (SD)	3.6 (3.2)	1.27 (1.7)	0.51	3.6 (3.2)	1.27 (1.7)	0.51	-	-	
	Min-Max	0–11	0–5		0–11	0–5		-	-	
missing	Mean (SD)	0.53 (1.46)	0	1	0.53 (1.46)	0	1	-	-	
	Min-Max	0–5	0		0–5	0		-	-	
filled	Mean (SD)	1.27 (2.12)	2.33 (2.02)	0.44	1.27 (2.12)	2.33 (2.02)	0.44	-	-	
	Min-Max	0–8	0–5		0–8	0–5		-	-	
GI	Mean (SD)	0.25 (.34)	0.24 (.27)	0.75	0.21 (.34)	0.19 (.29)	0.92	0.29 (.34)	0.28 (.25)	0.75
	Min-Max	0–1	0–1		0–1	0–1		0–1	0–0.83	
OHI-S	Mean (SD)	0.95 (.55)	0.85 (.55)	0.45	1.07 (.42)	0.90 (.62)	0.24	0.85 (.63)	0.81 (.50)	0.89
	Min-Max	0–2.17	0–2.17		0–1.83	0–2.17		0–2.17	0–1.50	

Using Mann–Whitney test

As much as 33.33% of patients and only 6.06% of children in the control group reported the occurrence of oral dryness during the day (*p =* 0.011) and significantly more frequently complained about the symptoms of angulitis (42%) compared with the control group (3%), *p =* 0.0002. 21.21% of patients reported the occurrence of occlusal abnormalities in the family whereas in the control group no occlusal abnormalities in the family occurred, *p =* 0.012. The children who did not suffer from JIA significantly more frequently consumed sweet snacks, as much as 64.29% reported the consumption of sweets more frequently than once a day whereas in the group of JIA children this percentage was only 24.24%, *p =* 0.009.

**Fig. 1:**
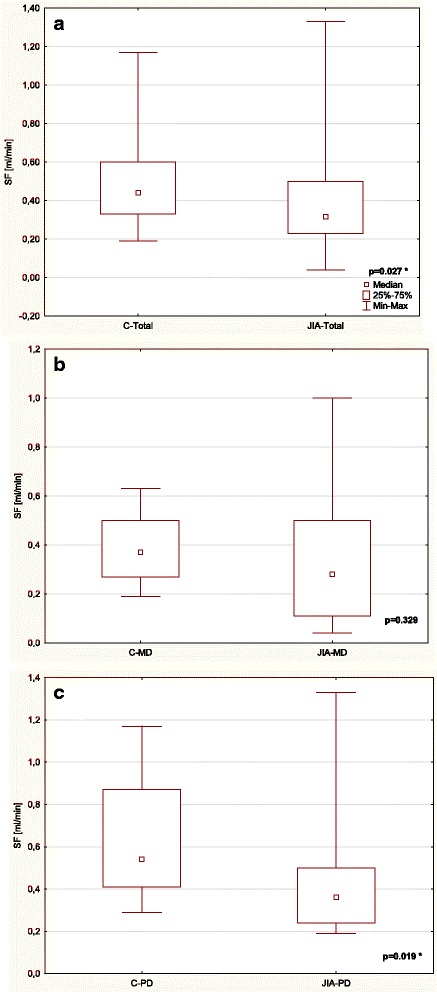
Comparison of SF in the UWS of JIA patients and controls (C) (**a**) including mixed (MD) (**b**) and permanent dentition (PD) (**c**). * statistically significant, *p <* 0.05

**Fig. 2:**
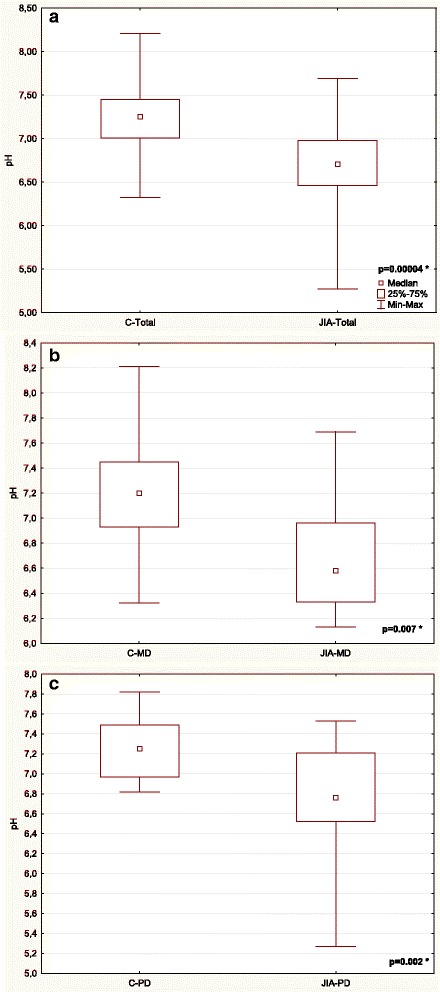
Comparison of pH in the UWS of JIA patients and controls (C) (**a**) including mixed (MD) (**b**) and permanent dentition (PD) (**c**). * statistically significant, *p <* 0.05

**Fig. 3:**
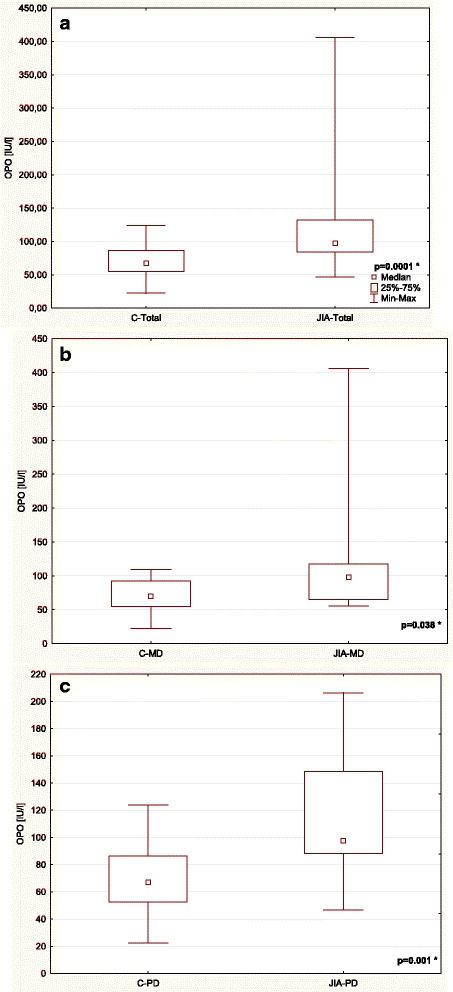
Comparison of the salivary OPO activity in the UWS of JIA patients and controls (C) (**a**) including mixed (MD) (**b**) and permanent dentition (PD) (**c**). * statistically significant, *p <* 0.05

**Fig. 4:**
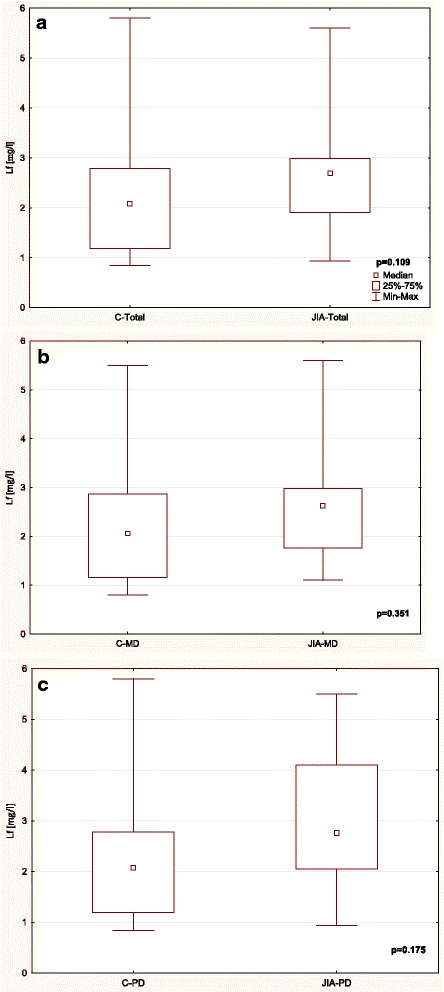
Comparison of Lf concentration in the UWS of JIA patients and controls (C) (**a**) including mixed (MD) (**b**) and permanent dentition (PD) (**c**).

**Fig. 5:**
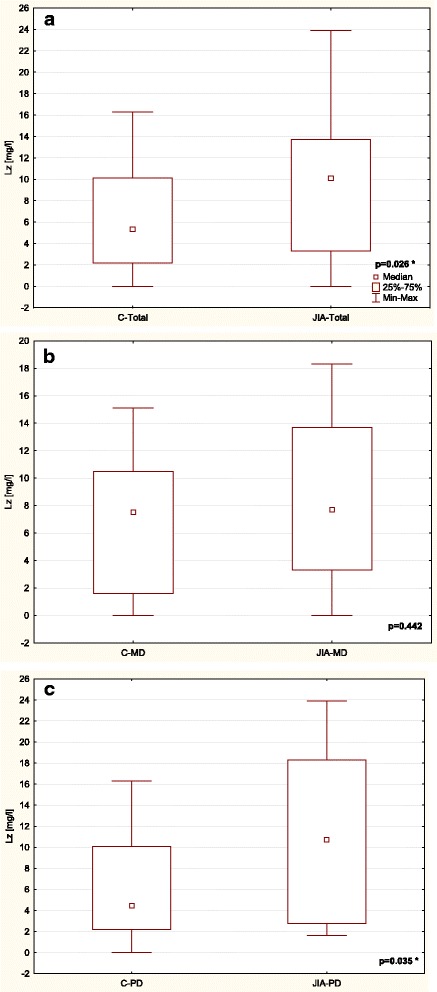
Comparison of Lz concentration in the UWS of JIA patients and controls (C) (**a**) including mixed (MD) (**b**) and permanent dentition (PD) (**c**). * statistically significant, *p <* 0.05

**Fig. 6:**
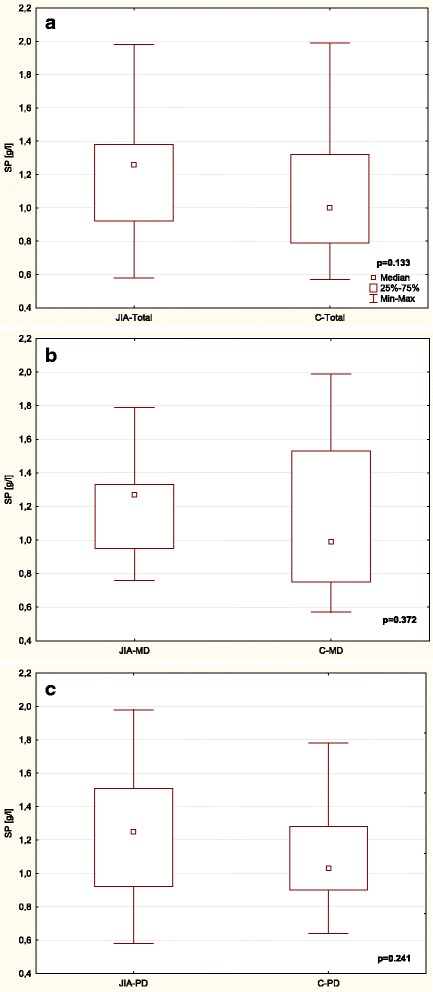
Comparison of SP concentration in the UWS of JIA patients and controls (C) (**a**) including mixed (MD) (**b**) and permanent dentition (PD) (**c**).

Table 3 and Figs. 1, 2, 3, 4, 5 and 6 show unstimulated SF, pH, OPO activity and Lz, Lf and SP concentrations in JIA children and control group including MD and PD. SF was significantly lower in the JIA group as compared with the C (*p =* 0.027), Fig. 1a. The classification of JIA children according to the type of dentition showed a significantly lower SF in the PD JIA group in comparison to the controls (*p =* 0.019), Fig. 1c. JIA children presented a significantly lower salivary pH than the control group (*p =* 0.00004), Fig. 2a. The division of patients according to the type of dentition also showed a similar pattern, a significantly lower pH was observed in both MD and PD JIA groups in comparison to the C (*p =* 0.007 and *p =* 0.002, respectively), Fig. 2b-c. The specific activity of OPO in the UWS of JIA patients was significantly higher than in the control group (*p =* 0.0001), Fig. 3a. A similar pattern was also found in JIA children with MD and PD in comparison to the C (*p =* 0.038 and *p =* 0.001, respectively), Fig. 3b-c. There were no significant differences in Lf concentrations in the UWS between the JIA and C groups either in the whole sample group or after the division of the children according to the type of dentition, Fig. 4a-c. The Lz concentration in the UWS of JIA patients was significantly higher than in the C group (*p =* 0.026), Fig. 5a. This pattern was also found in JIA children with PD in comparison to the C (*p =* 0.035), Fig. 5c. There were no significant differences in SP concentrations in the UWS between the JIA and the C groups, Fig. 6a-c.

**Table 3:** Salivary flow rate (SF), pH and selected salivary proteins of innate immune system in JIA children and controls (C) including mixed and permanent dentition

Salivary parameters		Total			Mixed dentition			Permanent dentition		
		JIA *N =* 34	C *N =* 34	*p-* value	JIA *N =* 15	C *N =* 15	*p-* value	JIA *N =* 19	C *N =* 19	*p-* value
SF [ml/min]	Mean (SD)	0.41 (.28)	0.51 (.25)	0.027	0.37 (.30)	0.38 (.14)	0.33	0.43 (.27)	0.61 (.27)	0.019
	Min-Max	0.04–1.33	0.19–1.17		0.04–1	0.19–0.63		0.19–1.33	0.29–1.17	
pH	Mean (SD)	6.74 (.52)	7.25 (.37)	0.00004	6.7 (.49)	7.21 (.45)	0.007	6.76 (.56)	7.28 (.31)	0.002
	Min–Max	5.27–7.69	6.32–8.21		6.13–7.69	6.32–8.21		5.27–7.53	6.82–7.82	
OPO activity [IU/ml]	Mean (SD)	112.72 (63.62)	70.03 (25.2)	0.0001	112.98 (84.86)	71.83 (24.11)	0.038	112.5 (42.65)	68.61 (26.6)	0.001
	Min-Max	46.72–406.1	22.33–123.68		55.7–406.1	22.33–109.31		46.72–206.38	22.33–123.68	
Lf [mg/l]	Mean (SD)	2.90 (1.46)	2.39 (1.49)	0.11	2.81 (1.51)	2.42 (1.55)	0.35	2.97 (1.46)	2.37 (1.48)	0.17
	Min-Max	0.94–5.6	0.8–5.8		1.1–5.6	0.8–5.5		0.94–5.5	0.84–5.8	
Lz [mg/l]	Mean (SD)	9.95 (6.76)	6.3 (4.73)	0.026	8.46 (5.76)	6.63 (4.92)	0.44	11.12 (7.4)	6.05 (4.7)	0.035
	Min-Max	0–23.9	0–16.3		0–18.3	0–15.1		1.62–23.9	0–16.3	
SP [g/l]	Mean (SD)	1.24 (.36)	1.11 (.39)	0.13	1.23 (.3)	1.14 (.47)	0.37	1.25 (.42)	1.09 (.33)	0.24
	Min-Max	0.58–1.98	0.57–1.99		0.76–1.79	0.57–1.99		0.58–1.98	0.64–1.78	

Using Mann–Whitney test

The specific activity of OPO in the UWS was inversely correlated with the GI index, whereas the salivary Lz concentration was inversely correlated with the dmft index in JIA patients (at the limit of statistical significance), Table 4. There was a positive correlation between the salivary pH and the OHI-S index in the control group, Table 4.

**Table 4:** Correlations between salivary parameters and oral health condition in JIA and control group (C)

Salivary parameters		JIA				C			
		DMFT	dmft	GI	OHI-S	DMFT	dmft	GI	OHI-S
SF [ml/min]	r	0.19	0.16	0.26	0.01	0.33	0.16	0.18	0.04
	p	0.269	0.557	0.132	0.960	0.060	0.558	0.302	0.810
pH	r	0.07	0.05	0.09	0.06	0.12	0.40	0.10	0.35
	p	0.693	0.863	0.592	0.739	0.498	0.136	0.572	0.042 *
OPO activity [IU/l]	r	−0.02	0.04	-0.41	0.08	−0.18	0.26	−0.28	0.11
	p	0.917	0.888	0.017 *	0.659	0.297	0.355	0.103	0.542
Lf [mg/l]	r	0.07	0.06	−0.01	0.09	−0.15	0.03	−0.18	−0.26
	p	0.678	0.824	0.937	0.628	0.404	0.923	0.296	0.142
Lz [mg/l]	r	−0.10	-0.51	−0.11	−0.11	−0.25	−0.08	0.09	0.24
	p	0.565	0.053 **	0.514	0.540	0.157	0.788	0.604	0.172
SP [g/l]	r	−0.03	−0.22	0.15	0.17	0.01	0.04	−0.15	−0.10
	p	0.883	0.427	0.403	0.345	0.952	0.883	0.401	0.561

Using Spearman’s rank correlation; *statistically significant, *p <* 0.05; **at the limit of statistical significance, *p =* 0.05

## Discussion

Saliva conditions the homeostasis in the oral cavity. Degenerative changes and the damage to structure and function of salivary glands occurring in the course of JIA [23, 39] may lead to a reduction in salivary flow and a change in physical properties of saliva. Our own studies proved a significant reduction of the resting salivary flow rate in the group of children with full permanent dentition, aged 12–18 years, suffering from JIA. A minimum resting salivary flow in the patients was only 0.04 ml/min. In as many as 8 patients the salivary flow was below 0.2 ml/min, which pointed to hyposalivation [40]. In addition, JIA patients significantly more frequently reported subjective complaints of oral dryness compared to the control group. It is probable that subjective complains of oral dryness in the sample population appeared before the occurrence of a reduced salivary flow (SF). Despite the literature describing populations of JIA children in whom no differences in the salivary flow rate compared to health children were found [24–26], most research studies have shown, similar to our presented own studies, a reduced salivary flow in JIA children [21–23, 41].

In the course of JIA occurs an increase of salivary viscosity which depends on the presence of glycoproteins, in particular mucins [42]. Thick and viscous saliva facilitates the deposition of bacterial plaque, which creates the conditions for the development of dental caries and may influence the oral health. In our studies, however, a significantly lower resting SF in the sample group of JIA patients did not co-occur either with a worse oral hygiene or with gingivitis compared to the control group. The findings of many studies differ markedly, some researchers have not found any differences in oral hygiene between the JIA patients and the control population [13, 18, 41, 43], whereas other studies have shown a worse oral hygiene in the course of JIA [14, 24, 25, 44, 45]. In contrast to the obtained results, also the co-occurrence of a higher accumulation of dental biofilm with a normal SF in JIA children compared to the control population has been described in the literature [24, 25]. The lack of coherence with previous studies could be explained by the fact that the SF is only one of factors which may modify the plaque accumulation in the course of JIA. The oral hygiene level strictly depends on daily preventive care.

According to some studies, gingivitis frequently occurs in children suffering from a rheumatic disease [18, 25, 41, 43, 45]. A higher intensity of gingivitis in JIA patients with a dental plaque accumulation similar to healthy individuals may result from the immunological origin of the disease [18, 41, 43]. However, we have not confirmed this association. Our own findings have shown the lack of differences in the occurrence of gingivitis between the JIA children and the control group, similar to other studies [13, 24, 46]. In the present study, a small gingival index seems to be a reflection of good oral hygiene. We can find an analogy to the study on adults with rheumatoid arthritis where a better periodontal status was found in RA patients than in controls [47].

A reduced salivary flow influences the chemical composition and the reaction of saliva. This study revealed altered biochemicals involved in the antimicrobial activity and the remineralisation of UWS in JIA patients. JIA patients have a lower salivary pH and a buffer capacity at pH ranges ≤7.0, which suggests these patients’ difficulty in maintaining a neutral pH [24]. Our own studies have proved a significantly lower pH of unstimulated saliva in the JIA patient group. The mean pH value was 6.73, whereas the minimum pH value was 5.27. JIA patients may have a favourable osmotic gradient for the loss of minerals from the tooth structure. The study of Walton et al. did not reveal any significant differences in pH of resting or stimulated saliva, but proved that the change in pH from unstimulated to stimulated was significantly lower in the JIA patients than in the control group [23].

Salivary OPO, an antioxidant enzyme, plays an important role in the inhibition of the microbiologic growth of bacteria, viruses and fungi and also reduces the cell toxicity in the oral cavity. Our own findings showed that the JIA children exhibited a major increase in antioxidant OPO activity in UWS. In addition, we described a significant negative correlation of peroxidase with the gingival index, which with good oral hygiene additionally explains the satisfactory gingival status in the sample population of JIA children. Both a significantly higher OPO concentration [27, 28] and a lack of differences in the OPO activity in UWS in JIA children [21] were reported in the literature. A reduction in the salivary OPO activity was reported by de Oliviera Perestrelo et al. [44]. Since the studies on the OPO activity concerned both unstimulated and stimulated saliva, they are difficult to compare.

Lz is indicative of the innate immune response in the oral cavity and acts as one of the microbiologic defence mechanisms. The enzyme is a strong cationic cell-lysing agent that can cause the lysis of oral bacterial cells. Our study revealed that JIA children with permanent dentition were characterized by a significantly higher salivary Lz level, which was in contrast with previously published studies [21, 26]. In our own studies, the SP concentration in saliva of JIA children was similar to the control group, so the significantly higher Lz concentration in JIA patients most probably resulted from an increased Lz production in JIA children. This result was additionally confirmed by the negative correlation of Lz with the mean dmft index which was found in children with mixed dentition. However, the strength of this correlation was average, which means that Lz is not the only factor which influences the caries intensity in deciduous teeth.

Lf is a transferrin protein which, due to a high affinity to iron ions, blocks the accessibility of iron for the bacteria. Lf demonstrates bactericidal, antiviral and antifungal action. In the course of JIA, no changes in the Lf concentration in UWS either in children aged 6–13 years or 11–18 years were observed. It is the first study assessing the Lf level in young JIA patients. The lack of data in available literature makes it impossible to directly refer to the level of this protein in other populations of JIA patients.

We did not observe any significant differences in the dmft or DMFT between the JIA children and the controls. It should be stressed that in the conducted study a very high intensity of dental caries in both JIA and C groups was found, however the mean dmf/DMF in JIA children and in controls were similar to epidemiological data from the subjects’ home region [48, 49]. Studies concerning dental caries in JIA children present contradictory results, showing either an increase in prevalence [18, 26] or no difference [22, 24, 41, 43, 45] compared with healthy individuals. An increase of caries intensity in JIA children was explained by a restricted mouth opening and an inflammatory condition of upper extremity joints, causing pain and difficulties in plaque removal [17, 18, 43]. These signs were not present in JIA children included in this study. According to the literature, the risk of caries may also be influenced by a frequent consumption of sweet snacks by JIA patients and a long-term pharmacotherapy with sugar-rich preparations which, especially when administered at night when the resting SF is minimal, remain in the oral cavity for a long time as a substrate for microorganisms causing dental caries [16, 50], however, we observed that the subjects significantly less frequently consumed sweets than the control group. Therefore, it seems that in our study the caries intensity in subjects and controls may have different origins – salivary disorders in JIA patients, proven by a lower pH and an improper diet in C. It has not yet been raised in the literature that the selection of a control group in studies on JIA patients should also take the diet as a variable into consideration. So in the case of caries the multicausation of the disease is to be taken into consideration [51].

Case control study is a type of research method useful in describing rare diseases, but it has some limitations [52]. Choosing a control group may be influenced by a selection bias. In our study, the controls came from one dental clinic; therefore, it could not be referenced for the entire population. The exclusion of early caries lesions could also be considered as a limitation of our study. In our research, similar to most studies presented in the literature, we concentrated on the analysis of UWS, which is a certain limitation of this study. The results of the study on stimulated saliva conducted by Melo et al. and Perestrelo et al. are also available [24, 44]. A full picture of changes occurring in saliva of JIA children could be given after the analysis of resting and stimulated saliva.

In our study, we showed the oral status and the saliva parameters as well as their mutual correlations for the entire sample group of children in a broad age bracket as well as divided into younger children with mixed dentition and children with full permanent dentition. The detailed analysis of obtained results depending on the age of the children seems to be interesting because the changes in saliva composition or salivary flow rate most frequently concerned children after 11 years of age, and only in rare cases occurred already in the group of children aged 6–10 years. To the best of our knowledge apart from the study of Welbury et al., we are the only authors who presented such detailed picture of the population of JIA children divided into age and type of dentition [45]. Based on an early detection of JIA and a separation of patients with a reduced salivary flow, it will be possible to take appropriate prevention and treatment measures. Paediatric dentists should be familiar with the symptoms, complications and oral manifestations of JIA to help manage the disease and provide quality care to these patients.

## Conclusions

In the course of JIA occur a reduction of the resting salivary flow rate and a decrease of saliva pH. In spite of this, no differences in the clinical oral status between the analysed JIA children population and the control group were found. The mobilisation of salivary proteins of the innate immune system, such as salivary peroxidase and lysozyme, contributes to the maintenance of healthy oral tissues.

## Abbreviations

C: Children not affected by JIA
dmft: Decayed, missing, filled deciduous teeth
DMFT: Decayed, missing, filled permanent teeth
ELISA: Enzyme-linked immunosorbent assay
GI: Gingival index
ILAR: International League of Associations for Rheumatology
JIA: Juvenile Idiopathic Arthritis
Lf: Lactoferrin
Lz: Lysozyme
MD: Mixed dentition
OHI-S: Oral hygiene index – simplified
OPO: Peroxidase
PD: Permanent dentition
RA: Rheumatoid Arthritis
RF: Rheumatoid factor
SF: Salivary flow rate
SP: Total salivary protein content
TMJ: Temporomandibular joint
UWS: Unstimulated whole saliva
